# Cardioneuroablation guided by real-time spectral analysis: The Omnipolar Technology Near Field

**DOI:** 10.1016/j.hrcr.2024.08.027

**Published:** 2024-09-01

**Authors:** Andrea Giomi, Andrea Bernardini, Alessandro Paoletti Perini, Cristiano Salvatore Zaccaria, Margherita Padeletti, Massimo Milli

**Affiliations:** 1 Cardiology and Electrophysiology Unit, Department of Medical Specialties, Azienda USL Toscana Centro, Santa Maria Nuova Hospital, Florence, Italy; 2 Department of Experimental and Clinical Medicine, University of Florence, Florence, Italy

**Keywords:** Cardioneuroablation, Ganglionated plexi, Spectral analysis, Syncope

**Key Teaching Points**

• Many different approaches to perform cardioneuroablation (CNA) have been proposed, but each method has its own limitation and no one has proved to be superior to the others.
• Omnipolar Technology Near Field (OTNF; Abbott, Plymouth, MN) is a novel mapping algorithm integrated into the EnSite X (Abbott) mapping system that allow a real-time automatic spectral analysis.
• The algorithm generates a frequency map, which depicts the distribution of high-frequency activity.
• In our case, OTNF algorithm was used to analyze the atrial substrate in a patient undergoing CNA, allowing the location of ganglionated plexi.
• This tool may help electrophysiologists to selectively target ganglionated plexi in the setting of a CNA procedure, avoiding imprecise or overextensive ablation.
• OTNF allows fast and accurate visualization of high-frequency atrial areas on a high-density electroanatomic map. This tool may help electrophysiologists to selectively target ganglionated plexi in the setting of a CNA procedure, avoiding imprecise or overextensive ablation.

## Introduction

Cardioneuroablation (CNA) is an emerging technique that aims to block the postganglionic vagal fibers of the heart, correcting the imbalance between sympathetic and parasympathetic tone. CNA has been proposed as an alternative to pacemaker implantation in patients with syncope when a cardio-inhibitory mechanism is documented.^1^^,^^2^ Many different approaches to performing CNA have been proposed in the past 2 decades; however, multiple topics still remain unclear. Among these, the localization of ganglionated plexi (GPs) is a priority issue. In previous papers, Pachón and colleagues^2^^,^^3^ reported that the spectrum of atrial electrograms (EGMs) can be used to identify areas of neuromyocardial interface and proposed that approach to guide CNA. Spectral analysis (SA) of atrial potentials was mostly used during the early phase of CNA investigation by a single group and has not achieved widespread diffusion, mainly because it requires dedicated equipment. Omnipolar Technology Near Field (OTNF; Abbott, Plymouth, MN) is a novel mapping algorithm integrated into the EnSite X (Abbott) mapping system, which allows real-time automatic SA with high spatial resolution and accuracy. In the present case, we describe the use of OTNF to create a frequency map, leading the CNA procedure.

## Case report

**Figure 1:**
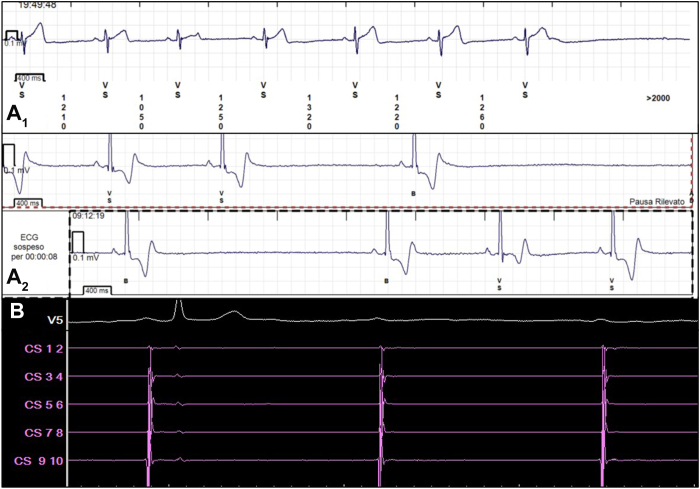
**A**_**1**_**, A**_**2**_**:** Implantable loop recorder events of vagal atrioventricular (AV) block and sinus node arrest resulting in bradycardia and symptomatic events; A_1_ and A_2_ events were recorded during daytime. **B:** Spontaneous vagal AV block recorded during an early phase of electrophysiological study.

**Figure 2:**
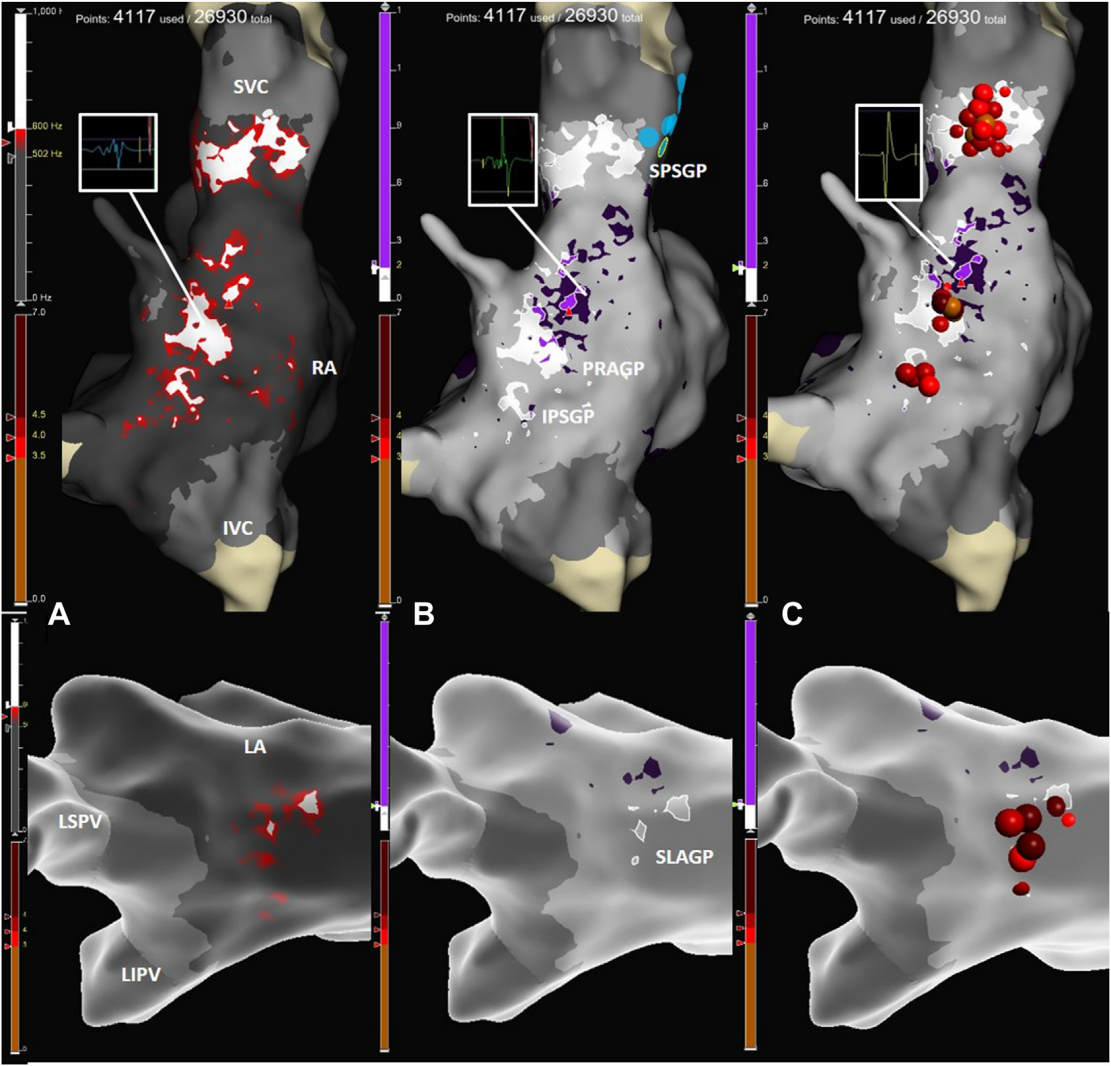
*Upper panels:* Right atria (RA) shown by a posterior view; *lower panels:* left atria (LA) shown by cranial view. **A:** Frequency map by EnSite X Omnipolar Technology Near Field (OTNF): high-frequency zones are shown in *white*, low-frequency areas are shown in a *darker gray*, transitional zone in *red*. **B:** Superimposition of frequency and fragmentation map: high-frequency zones are shown in *white* and high fragmentation ones are in *purple*. A mismatch between the areas of high frequency and high fragmentation can be detected in posterior right atrial ganglionated plexi (PRAGP) and superior left atrial ganglionated plexi (SLAGP). **C:** Ablation tags on (from *top* to *bottom*): 1 = superior paraseptal ganglionated plexi (SPSGP); 2 = PRAGP; 3 = inferior paraseptal ganglionated plexi (IPSGP); 4 = SLAGP. *White boxes*: representative electrograms of high-frequency area, high-fragmentation area, and normal myocardium. IVC = inferior vena cava; LIPV = left inferior pulmonary vein; LSPV = left superior pulmonary vein; SVC = superior vena cava.

A 47-year-old man with recurrent vaso-vagal syncopal episodes, despite lifestyle modification and physical counterpressure maneuvers, was referred to our hospital for general assessment. Basal 12-lead electrocardiogram showed sinus bradycardia (resting heart rate 40 beats/min) and long PR (230 milliseconds) without other pathologic findings. Transthoracic 2-dimensional echocardiography was negative for structural heart abnormalities. An implantable loop recorder was implanted to allow continuous heart rhythm monitoring. Immediately after the implant, several episodes of sinus arrest, advanced atrioventricular block, and second-degree atrioventricular block Mobitz type II events were detected, inclusive of a prolonged daytime event of sinus bradycardia with a P–P interval of 4 seconds, resulting in presyncope (Figure 1). Considering the young age of the patient, the CNA opportunity was discussed with the patient as an alternative treatment to pacemaker implantation. A basal electrophysiological (EP) study was performed after providing mild sedation: baseline EP intervals resulted normal (PP 1342 milliseconds, AH 100 milliseconds, HV 50 milliseconds, corrected sinus node recovery time 200 milliseconds). Transient complete atrioventricular block episodes were observed during the early phase of the EP study as a response to painful groin manipulation (Figure 1B). Incremental atrial pacing protocols ruled out infra-Hisian blocks and confirmed an intra-nodal block (Wenckebach point 780 milliseconds, 77 beats/min). A multipolar mapping catheter (Advisor HD Grid, Abbott) was introduced in the right atrium and an electroanatomic map was acquired during sinus rhythm on the EnSite X (Abbott) platform. To map the left atrium, the mapping catheter was advanced through a patent foramen ovale. Ultra-high-density mapping of both the atria was ensured by a total of 4117 points. As a first step to localize GPs areas, a fragmentation bipolar map was created, using a threshold of 2 deflections at a filtering setting of 200–500 Hz, as proposed by Aksu and colleagues.^4^ A second frequency map was then created using the OTNF by adjusting the frequency window to maximize the isolation of high-frequency areas. Peak frequency was set at 600 Hz to highlight the smallest areas with high-frequency spectrum in the anatomic GP positions. A mismatch between frequency and fragmentation maps was observed in the superior paraseptal GP and superior left atrial GP, where high-frequency potentials did not show equally high fragmentation (Figure 2). Areas with high-frequency spectrum were accepted as potential GP sites and treated with radiofrequency ablation. After tracking the right phrenic nerve course by high output pacing, radiofrequency applications were delivered using the 3.5-mm tip ablation TactiCath catheter (Abbott) in the OTNF highlighted areas, following this order: superior paraseptal GP, inferior paraseptal GP, posterior right atrial GP through right atrium, superior left atrial GP, posterolateral left atrial GP, posterior right atrial GP (through left atrium). Ablation was guided by the Lesion Index and local potential elimination. If a vagal response was evidenced during ablation, further radiofrequency applications were delivered until the complete disappearance of the vagal response. Systemic anticoagulation with heparin was maintained during left atrium ablation with an active clotting time target between 250 and 350 seconds. Once all GP areas had been ablated, significant changes in EP intervals were observed: PP and PR intervals decreased at 770 milliseconds and 180 milliseconds, respectively, and Wenckebach point increased at 580 milliseconds. A bolus of 2 mg of atropine obtained a variation of PP interval of just 3% (746 milliseconds), demonstrating complete vagal denervation. The patient was monitored for the following 24 hours by continuous electrocardiography. Heart rate constantly >75 beats/min was maintained for all the following hospitalization, and no atrioventricular blocks were detected. A brief follow-up of 1 month provided by implantable loop recorder did not evidence recurrence of bradycardia, sinus arrests, or atrioventricular blocks.

## Discussion

This clinical case focused on the utility of OTNF technology to locate GPs during a CNA. This tool is integrated into the Abbott EnSite X platform and provides information about the spectrum of frequencies of each electrogram. Although the algorithm's primary application is to improve discrimination between far-field and near-field potentials, it can also be used for other purposes.

The importance of SA in identifying neuromyocardial interface was first described by Pachón and colleagues back in 2004.^5^ By studying the spectrum of the endocardial potentials by fast Fourier transformation, they found that some atrial areas present a fibrillar pattern characterized by a wide spectrum of frequencies with multiple components >100 Hz.^5^ The authors postulated that the fibrillar spectrum was the result of a heterogeneous composition of atrial myocardium where nervous fibers penetrating the atrial wall interfere with cardiomyocyte conduction. However, the rest of the atrial myocardium, called “compact,” displays a sharp spectrum of frequencies with a main component approximately 40 Hz reflecting homogenous substrate and uniform conduction. Several observations led to the conclusion that areas of fibrillar myocardium, also called “AF nests,” were distributed in the anatomic position of GPs and presented vagal responses at radiofrequency, although representing a good marker of neuromyocadial interface.^3^^,^^6^^,^^7^ Ablation protocols targeting these areas showed good results in achieving acute complete denervation and preventing recurrences in patients with neurally mediated syncope.^1^, ^2^, ^3^^,^^8^ However, SA has never been performed as a “stand-alone” strategy and its spread to the rest of the EP community has been limited, mostly because it requires dedicated equipment and specific skills that are not available in most EP laboratories. Other modalities to target GPs are considered more reproducible and can be found in the majority of studies on CNA. Among these, high-frequency stimulation,^9^ anatomic ablation,^10^^,^^11^ and fractionation mapping^12^ are the main ones. Each method has its own limitation and no one has proved to be superior to the others.

**Figure 3:**
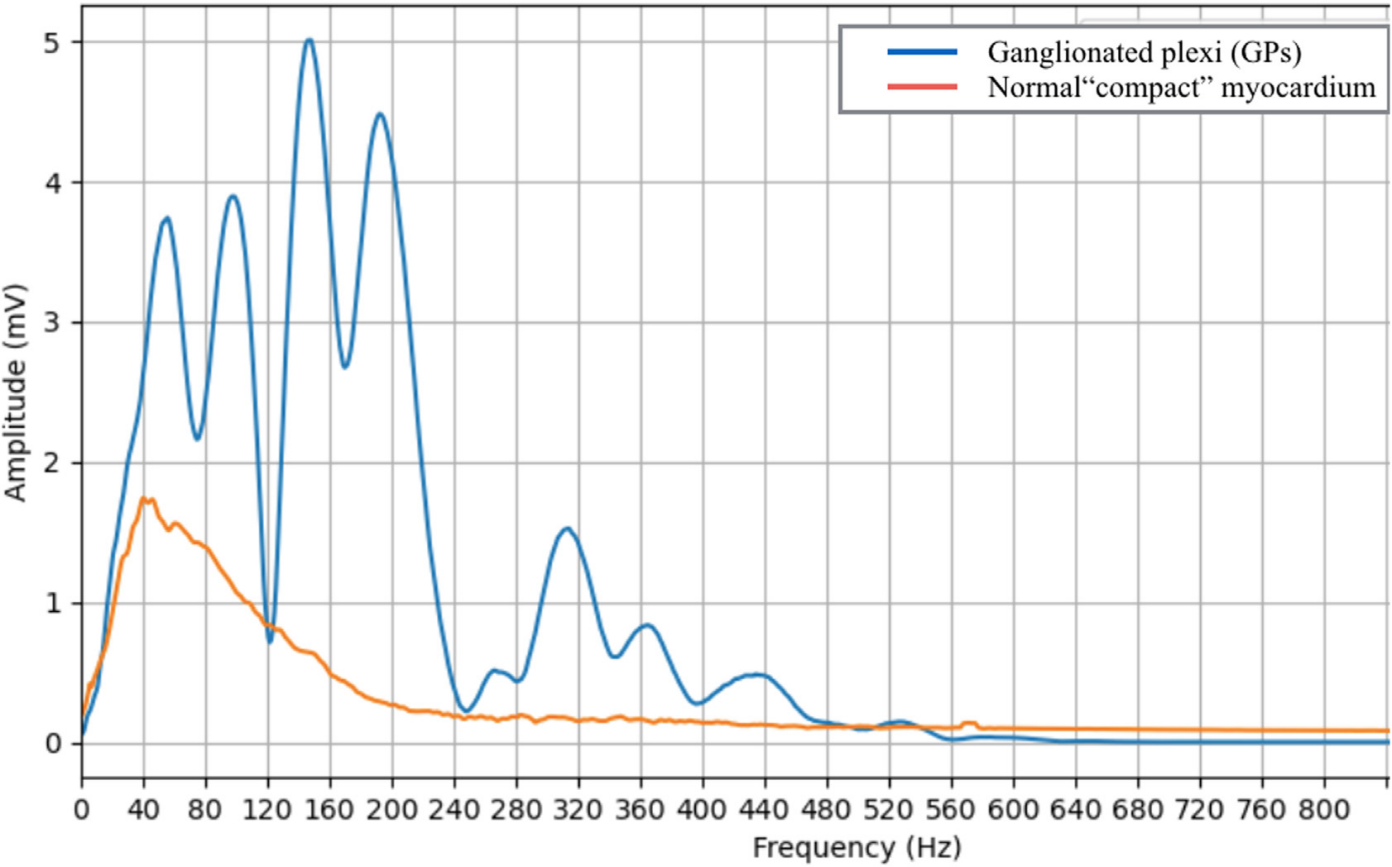
Example of spectrograms of ganglionated plexi (GPs) signal (*blue line*) and normal compact myocardium signal (*red line*). The compact myocardium registered in the septal region of right atrium presents a homogeneous spectrum with main frequencies at 40–60 Hz, while the electrogram in the GP area presents a wide spectrum with multiple components up to 600 Hz.

EGM fragmentation and high-frequency spectrum are probably 2 faces of the same phenomenon, although their relationship has never been clarified. Many data support that fragmentation of EGM is a functional property related to underlying rhythm and wavefront propagation.^13^^,^^14^ However, the definition of fractionated atrial EGM is still under debate both in sinus rhythm and in atrial fibrillation.^1^^,^^15^, ^16^, ^17^ Furthermore, mapping parameters like filter setting, width, refractory time, and fractionation threshold are critically important to define a fragmented bipolar potential, and are not standardized for GPs identification. SA of each atrial potential by fast Fourier transformation allows a whole acquisition of local information with minimal upstream filtering, consequently allowing the finest tissue characterization. However, in the absence of an automatic mapping tool, the main limitation of point-by-point examination would be longer procedural times and difficulty achieving a high spatial definition. In our opinion, the spectral-guided ablation proposed by Pachón and colleagues^2^^,^^3^ has the most reliable background and should be revisited in the light of the latest technologies. OTNF is an algorithm integrated into the EnSite X platform that evaluates signal frequency (Figure 3). The algorithm automatically measures and annotates the highest peak frequency associated with mapped intracardiac EGMs. This algorithm generates a frequency map, which depicts the distribution of high-frequency activity. On that map, high-frequency zones are shown in *white* and low-frequency areas are shown in a *darker gray*.

In our case, OTNF algorithm was used to analyze the atrial substrate in a patient undergoing CNA. The frequency map was obtained by adjusting frequency ranges to identify the smallest area with high frequency in the anatomic GPs position. *White areas* were detected in correspondence with 5 GPs areas. For the posterior-right atrial GP, high-frequency EGMs were detected from both the right and left atria. To better understand the relationship between SA and fragmentation, the frequency map was compared with a fragmentation map obtained from the same pool of points, using fragmentation settings proposed by Aksu and colleagues,^4^ and a mismatch was observed between the maps. In particular, the superior paraseptal GP and superior left atrial GP showed no fragmentation, despite a high frequency. For other GPs fragmentation and high-frequency areas were comparable. All *white areas* (high-frequency EGMs) underwent RF application until the complete elimination of local potential and suppression of any vagal response. After ablation, complete vagal denervation was reached and validated by atropine challenge. To our knowledge, this represents the first experience when OTNF technology has been exploited to allow real-time SA during CNA. This approach could overcome equipment and reproducibility issues that have limited SA among electrophysiologists. However, further comparative studies are needed to test the superiority of this technique over other GPs localization protocols.

## Conclusion

OTNF allows fast and accurate visualization of high-frequency atrial areas on a high-density electroanatomic map. This tool may help electrophysiologists to selectively target GPs in the setting of a CNA procedure, avoiding imprecise or overextensive ablation.

## Disclosures

The authors have no conflicts of interest to disclose.
